# Root-Associated Bacteria Community Characteristics of Antarctic Plants: *Deschampsia antarctica* and *Colobanthus quitensis*—a Comparison

**DOI:** 10.1007/s00248-021-01891-9

**Published:** 2021-10-18

**Authors:** Anna Znój, Jan Gawor, Robert Gromadka, Katarzyna J. Chwedorzewska, Jakub Grzesiak

**Affiliations:** 1 Department of Antarctic Biology, Institute of Biochemistry and Biophysics, Polish Academy of Sciences, Pawińskiego 5A, 02-106 Warsaw, Poland; 2 Botanical Garden-Center for Biological Diversity Conservation, Polish Academy of Sciences, Prawdziwka 2, 02-973 Warsaw, Poland; 3 Environmental Laboratory of DNA Sequencing and Synthesis, Institute of Biochemistry and Biophysics, Polish Academy of Sciences, Pawińskiego 5A, 02-106 Warsaw, Poland; 4 Department of Botany, Warsaw, University of Life Sciences-SGGW, Nowoursynowska 159, 02-776 Warsaw, Poland

**Keywords:** Functional symbiosis, Antarctic bacteria, Rhizosphere, Endosphere, Microbial diversity

## Abstract

*Colobanthus quitensis* (Kunth) Bartl. and *Deschampsia antarctica* Desv. are the only *Magnoliophyta* to naturally colonize the Antarctic region. The reason for their sole presence in Antarctica is still debated as there is no definitive consensus on how only two unrelated flowering plants managed to establish breeding populations in this part of the world. In this study, we have explored and compared the rhizosphere and root-endosphere dwelling microbial community of *C*. *quitensis* and *D*. *antarctica* specimens sampled in maritime Antarctica from sites displaying contrasting edaphic characteristics. Bacterial phylogenetic diversity (high-throughput 16S rRNA gene fragment targeted sequencing) and microbial metabolic activity (Biolog EcoPlates) with a geochemical soil background were assessed. Gathered data showed that the microbiome of *C*. *quitensis* root system was mostly site-dependent, displaying different characteristics in each of the examined locations. This plant tolerated an active bacterial community only in severe conditions (salt stress and nutrient deprivation), while in other more favorable circumstances, it restricted microbial activity, with a possibility of microbivory-based nutrient acquisition. The microbial communities of *D*. *antarctica* showed a high degree of similarity between samples within a particular rhizocompartment. The grass’ endosphere was significantly enriched in plant beneficial taxa of the family *Rhizobiaceae*, which displayed obligatory endophyte characteristics, suggesting that at least part of this community is transmitted vertically. Ultimately, the ecological success of *C*. *quitensis* and *D*. *antarctica* in Antarctica might be largely attributed to their associations and management of root-associated microbiota.

## Introduction

Antarctica is a place of extremes [1]. Only two species of *Magnoliophyta* (flowering plants) managed to establish their presence in this remote and life-challenging region: a member of the family *Poaceae*—*Deschampsia antarctica* Desv. (Antarctic hairgrass) and *Colobanthus quitensis* (Kunth) Bartl. (Antarctic pearlwort), belonging to the family *Caryophyllaceae* [2]. Their vast distribution in maritime and coastal Antarctica has baffled scientists for decades and was a subject of many debates [2–4]. Originally from South America, those two *Magnoliophyta* were suspected of being migratory relics from the Oligocene-Pliocene colonization event [2], while other data hints towards their more recent arrival during the late Pliocene [5]. Nonetheless, their ecological success in harsh Antarctic conditions is undisputed and attributable mostly to the extensive adaptations to many stress factors like cold, freeze–thaw cycles, UV radiation, drought, flooding, high concentration of sodium, and varying levels of nutrient concentrations from severe deficit to extreme over manuring [4]. To cope with environmental stresses, Antarctic plants have evolved adaptations in terms of molecular, cellular, and physiological functions to boost their cold tolerance by increasing their metabolic rates upon cold acclimation [6], such as stress protein production, upregulation of antioxidant synthesis, and accumulation of compatible solutes like amino acid proline and non-structural carbohydrates [7, 8] and secretion of antifreeze proteins into their apoplasts [9]. They also developed low temperature-efficient photosynthetic and respiratory systems whose maximal activity occurs at 13 °C, and 30% of its maximal photosynthetic capacity is retained at 0 °C [10, 11]. Moreover, such ontogenesis-related features of Antarctic flowering plants like long life cycles, an extended *primordium* development of leaves and flowers [12], or diverse propagation strategies based on seed production derived from self-pollination and less often cross-pollination (*C*. *quitensis* and *D*. *antarctica*) or vegetative reproduction (only *D*. *antarctica*) enabled continuous habitat range expansion of those plants since 1960, presumably aided by the ongoing global climate warming [13, 14]. However, the geographical and physical isolation of Antarctica still enforces low intra-species genetic diversity by restricting gene flow between Antarctica and South America into those populations [15, 16].

The relations between plants and microbes are now fairly well understood for the flora of temperate and tropical regions [17] but are somewhat under researched for polar regions, especially for Antarctica [18]. It is the consensus that microbial communities associated with the host plants’ roots exert the greatest influence on the plant health and development, most notably those that reside within two distinct rhizocompartments: the rhizosphere and the root endosphere [19]. Rhizospheric microbes inhabit the root-adjacent soil and feed on the root-derived organic exudates, whereas the endosphere community consists largely of plant-specific endosymbionts selectively recruited from the rhizosphere [20]. Some studies indicate that rhizospheric and endospheric microbial communities are not only spatially separated but also display different niche characteristics [21]. Rhizospheric bacteria are mostly engaged in mineral substrate dissolution by secreting organic acids (often gluconic acid), siderophores, or even cyanides consequently providing the plant with bioavailable phosphorus and biogenic metals. Their main role also includes biocontrol of soil-borne pathogens by competing with them for resources with the use of antimicrobial agents like bacteriocin-like peptides, different classes of antibiotics, and lytic enzymes: chitinase or β-1,3-glucanase. Rhizobacteria also have a direct input into root-growth stimulation by secretion of phytohormones such as indole-3-acetic acid. Endospheric bacteria on the other hand are engaged in plant stress responses mainly by reducing the inner-plant stress hormone ethylene levels through ACC deaminase-mediated hydrolysis. Endophytes also have a major contribution in plant-growth enhancement by providing fixed nitrogen, which is the main limiting element in soil environments [22].

As mentioned, studies on the Antarctic plant-associated microbiome (especially the bacterial part) are largely underrepresented in literature [23]. Available but scarce data allow us to draw only limited conclusions on microbiological phenomena connected to Antarctic *Magnoliophyta* [24–26]. This is largely due to the low resolution of the techniques used but also due to low sample diversity (mostly from only one particular site) or even exclusion of a vital part of the community, namely the endosphere, from analysis. Therefore, in this study, we employed cultivation-independent methods to compare root-associated microbial communities of *C*. *quitensis* and *D*. *antarctica* sampled from sites displaying contrasting edaphic, ecologic, and microclimatic characteristics. Our hypothesis states that the phylogenetic and metabolic diversity of microbial communities associated with native Antarctic plants is greater in sites of high stress-inducing factor intensity than in those providing milder growth conditions. To gain the necessary knowledge on the differences and similarities within the rhizospheric and endosphere microbial communities of Antarctic flowering plants, we made use of the high-throughput 16S rRNA gene fragment targeted sequencing (assessing the bacterial communities phylogenetic diversity) and community-level physiological profiling by Biolog EcoPlates.

## Materials and Methods

### Sites and Sampling

Samples were collected during the austral summer season of 2017–2018 from three sites on King George Island, South Shetland Islands, maritime Antarctica (Table 1). Several specimens (4–6 per site) were collected with the root adjacent soil with the use of sterile tools into sterile plastic containers and transported frozen (− 20 °C) to the laboratory in the Institute of Biochemistry and Biophysics, Polish Academy of Sciences (IBB, PAS). Additionally, bulk soil samples from those sites were gathered in triplicates (approximately 1.5 kg per site) for component analysis and transported in the same conditions.

**Table 1:** Sampling sites characteristics

	Site	Geographical coordinates	Distance to the sea	Altitude	Structure of vegetation	Landform and habitat
1	Lions Rump	62° 08′01″ S 58° 07′25″ W	100 m	1 m.a.s.l	Dense with crustose, fruticose and foliose lichens (60%), mosses (30%), isolated *Colobanthus quitensis* (5%), and *Deschampsia antarctica* (5%) specimens	Scree debris; Eutric Protic Skeletic Leptic Regosol (Turbic). Very limited human influence, remains under direct influence of marine aerosols
2	Puchalski Hill	62° 09′48″ S 58° 28′09″ W	500 m	110 m.a.s.l	Dense with *Colobanthus quitensis* (15%), *Deschampsia antarctica* (15%), mosses (20%), and fruticose and foliose lichens (50%)	Tundra on slope; Skeletic Protic Turbic Cryosol (Dystric, Humic, Ornithic). The site is located on an abandoned penguin colony, fertile, dry and exposed, with a little influence of marine aerosols
3	Point Thomas Penguin Rookery	62° 09′45″ S 58° 27′46″ W	100–120 m	10 m.a.s.l	Dense with *Deschampsia antarctica* (60%), mosses (15%), crustose lichens (15%), and *Colobanthus quitensis* (10%)	Bare rocks with soils enriched by penguins; Eutric Skeletic Lithic Leptosol (Humic, Ornithic, Protic). Area in the vicinity of breeding colony of penguins. Moist site supplied with water from melting snow with washings of guano deposits from penguin’s rookeries, remains under direct influence of marine aerosols

### Measurement of Soil Components

Soil pH (in 1 M KCl) and salinity (in double-distilled water) were measured with a CPC-411 Elmetron™ multiparameter probe [27]. Phosphates and nitrates were determined spectrophotometrically in a Shimadzu UV 1601 spectrophotometer and in an Epoll-Eco 20 spectrophotometer, respectively. Other elements were determined by atomic absorption spectroscopy [28].

### Bacterial Extraction

Bacterial cells were extracted from rhizospheric soil and roots samples from nine *C*. *quitensis* individuals (three per site) and respectively the same number of *D*. *antarctica* individuals. The following method was devised based on the findings of Lunau et al. [29] regarding the separation of prokaryotic cells from mineral and organic debris and the guidelines provided by Szymańska et al. [30] regarding root-associated microbe isolation. To analyze the microbiome of the root-adjacent soil, a sample of the soil was carefully removed from between the roots with a sterile spatula onto a pre-sterilized aluminums foil piece. Approximately 1 g of the soil was weighed and placed in a 50-mL conical tube containing 20 mL of sterile and cool (4 °C) dilution liquid composed of 0.9% (w/v) NaCl and 10 mM tetrasodium pyrophosphate (Na_4_P_2_O_7_). The suspension was then shaken for 30 min in a Tornado™ Vortexer at 2000 rpm at 4 °C. The tubes were then placed in a VWR Ultrasonic Cleaner USCTH filled with chilled water and sonicated for 60 s. The tubes were vortexed afterwards for 30 s to suspend detached cells. After brief centrifugation (1 min; 1000 rpm; 4 °C), the suspension was submitted to metabolic fingerprinting by the Biolog EcoPlate technique. To detach the rest of the adhering soil, the root system was washed in 60 mL of sterile NaCl/Na_4_P_2_O_7_ solution by shaking for 30 min in the aforementioned shaker (1000 rpm; 4 °C) and then rinsed 3 times in 5 mL of the same sterile and cooled solution by vortexing. Washed roots were sterilized by incubation in a cooled 10% hydrogen peroxide (H_2_O_2_) solution for 5 min and then rinsed 3 times with sterile NaCl/Na_4_P_2_O_7_ solution. The so surface-sterilized roots were placed in a pre-cooled sterile mortar. Two and a half milliliter of sterile NaCl/Na_4_P_2_O_7_ was added with 0.6 g of sterile, sharp garnet sand (lysing matrix A, MP Biomedicals) and gently ground with a pestle, allowing the sharp angular garnet pieces to comminute the roots to an amorphous pulp. The pulp was transferred to a 50-mL conical tube containing 20 mL of sterile and cool (4 °C) NaCl/Na_4_P_2_O_7_ solution and submitted to the above-mentioned procedure (shaking, ultrasonication, and vortexing). The resulting supernatant suspension was submitted to metabolic fingerprinting by the Biolog EcoPlate technique and DNA extraction. All reagents used in the bacterial extraction steps were molecular biology grade and were purchased from Sigma Aldrich.

### DNA Extraction

Rhizosphere soil DNA was extracted using the PowerSoil® DNA isolation kit (QIAGEN GmbH, Hilden, Germany) according to manufacturer protocol. An approximately 0.2 g of soil was used. DNA solutions were kept at 4 °C for further analysis. The dilution liquid containing endosphere bacteria was passed through a sterile 47-mm Whatman polycarbonate filter (0.22 µm pore size). The DNA from the filter-trapped bacteria was extracted using the PowerWater® DNA isolation kit (QIAGEN, GmbH, Hilden, Germany) according to manufacturer protocol and kept at 4 °C.

### 16S rRNA Gene Amplification

The phylogenetic study was performed by targeted sequencing and analysis of the prokaryotic 16S ribosomal RNA gene. A fragment of the 16S rRNA gene containing the V3 and V4 variable regions was amplified using gene-specific primers: 16S_V3-F and 16S_V4-R positions 341-357F and 785-805R, respectively, according to *Escherichia coli* 16S rRNA gene reference sequence [31]. Illumina Nextera XT overhang adapter nucleotide sequences were included in addition to the 16S rRNA gene-specific sequences, which allowed sample indexing and pooling. Each PCR was conducted in triplicates using KAPA HiFi PCR kit (Roche, Basel, Switzerland) in a final volume of 20 µL per reaction according to the manufacturer’s instructions.

### Amplicon Sequencing

Obtained PCR products were pooled into 12 samples (2 rhizocompartments × 3 sampling sites × 2 plant species) in equimolar ratio and indexed using Nextera XT barcodes (Illumina, San Diego, CA, USA). Amplicon libraries were sequenced on Illumina MiSeq instrument (Illumina, San Diego, CA, USA) in the DNA Sequencing and Oligonucleotide Synthesis Laboratory (IBB, PAS). Sequencing was conducted in paired-end mode (2 × 300 bp) with the use of a v.3 (600 cycles) chemistry cartridge, which allowed the generation of long paired reads fully covering 16S V3–V4 amplicons.

### Phenotype Fingerprinting with Biolog EcoPlate™

The Biolog EcoPlate assay determines the ability of a mixed microbial community to use any of 31 carbon compounds as the sole carbon source (plus a single control well with no-carbon). Microbial communities were characterized for their ability to catabolize 10 different carbohydrates, 9 carboxylic and acetic acids, 4 polymers, 6 amino acids, and 2 amines [32]. Root-associated bacterial suspensions were adjusted with sterile 0.9% NaCl to optical transmittance of 0.9. One hundred microliter aliquots of each suspension were added to each well of EcoPlate microplates (Biolog Inc., Hayward, CA, USA). The plates were incubated in darkness at 10 °C. The temperatures were chosen to accommodate the activity range of the resident microbial communities: the psychrophiles and the psychrotrophs [33]. The color development was read at 590 nm (A_590_) in a Varioskan plate reader (Thermo Fisher Scientific, Waltham, MA, USA), and cellular respiration was measured kinetically by determining the colorimetric reduction of tetrazolium dye. Data were collected approximately twice a week over a 65-day period. The prolonged incubation of EcoPlates was based on our previous observations [34–36]. Data from the 36th day of incubation were used as there was no further color development after this date. Final absorbance data were first blanked against the time zero reading and then blanked against the respective control well containing no carbon source. Readings that had the *A*_590_ value of 0.25 or higher were scored as a positive EcoPlate response (PER).

### Data Analysis

Raw sequencing data were cleaned, aligned, and classified automatically by the EzBioCloud platform using the PKSSU4.0 database [37]. Chimeric, low quality, and non-target (chloroplast, mitochondrial, and archaeal) amplicons were automatically discarded. The operational taxonomic unit was defined as a group of sequences that exhibit greater than 97% similarity to each other. Illumina reads were deposited in the NCBI Sequence Read Archive (SRA) as BioProject PRJNA726953. All results were compiled using Excel 2016 (MS Office) for Windows. A two-sample *t*-test was applied to compare different data sets. Variance within the sets was assessed using the *f*-test beforehand. Correlations between biological and geochemical parameters were calculated using Pearson’s correlation coefficient. Principal component analysis (PCA) was performed using the singular value decomposition method. Data visualization and statistical analysis have been performed using the R software (R v.4.0.2) and the following packages: ggplot2, fmsb, Hmisc, ggpubr, corrplot, and autoplot [38].

## Results

**Fig. 1:**
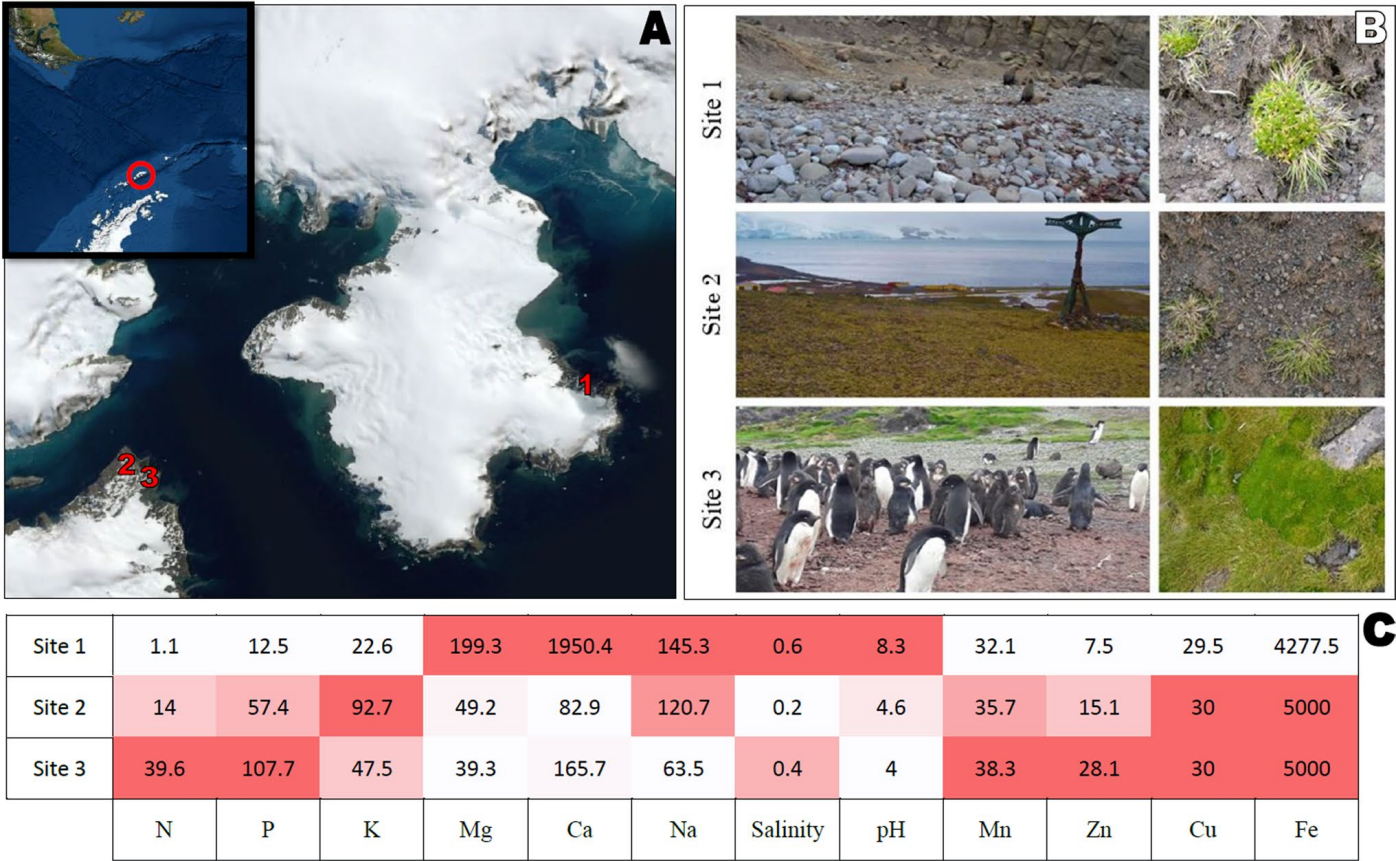
Sampling site details. **A** Satellite map displaying the geographical situation of the sampling sites: red circle, King George Island, Maritime Antarctica; 1, sampling site 1, Lions Rump, King George Bay shore; 2, Puchalski Hill; 3, Point Thomas Penguin Rookery, Admiralty Bay shore. **B** Sampling sites 1–3 landscape and ground photographs. **C** Sampling sites 1–3 geochemical composition of the soil; N, mg NO_3_/100 g soil; P, mg P_2_O_5_/100 g soil; K, mg K_2_O/100 g soil; Mg, mg Mg/100 g soil; Ca, mg Ca/100 g soil; Na, mg Na/100 g soil; salinity, g NaCl/L; Mn, mg Mn/kg soil; Zn, mg Zn/kg soil; Cu, mg Cu/kg soil; Fe, mg Fe/kg soil

Site 1 soil was characterized by high magnesium, very high calcium, and relatively high sodium contents and salinity, and also pH was high (8.3). Soil from site 2 had the highest potassium content among the examined soil samples with high copper and iron concentrations and a low pH (4.3). Site 3 soils had the highest concentration of nitrates, phosphates, manganese, and zinc with the lowest reported pH (4.0) (Fig. 1C).

**Fig. 2:**
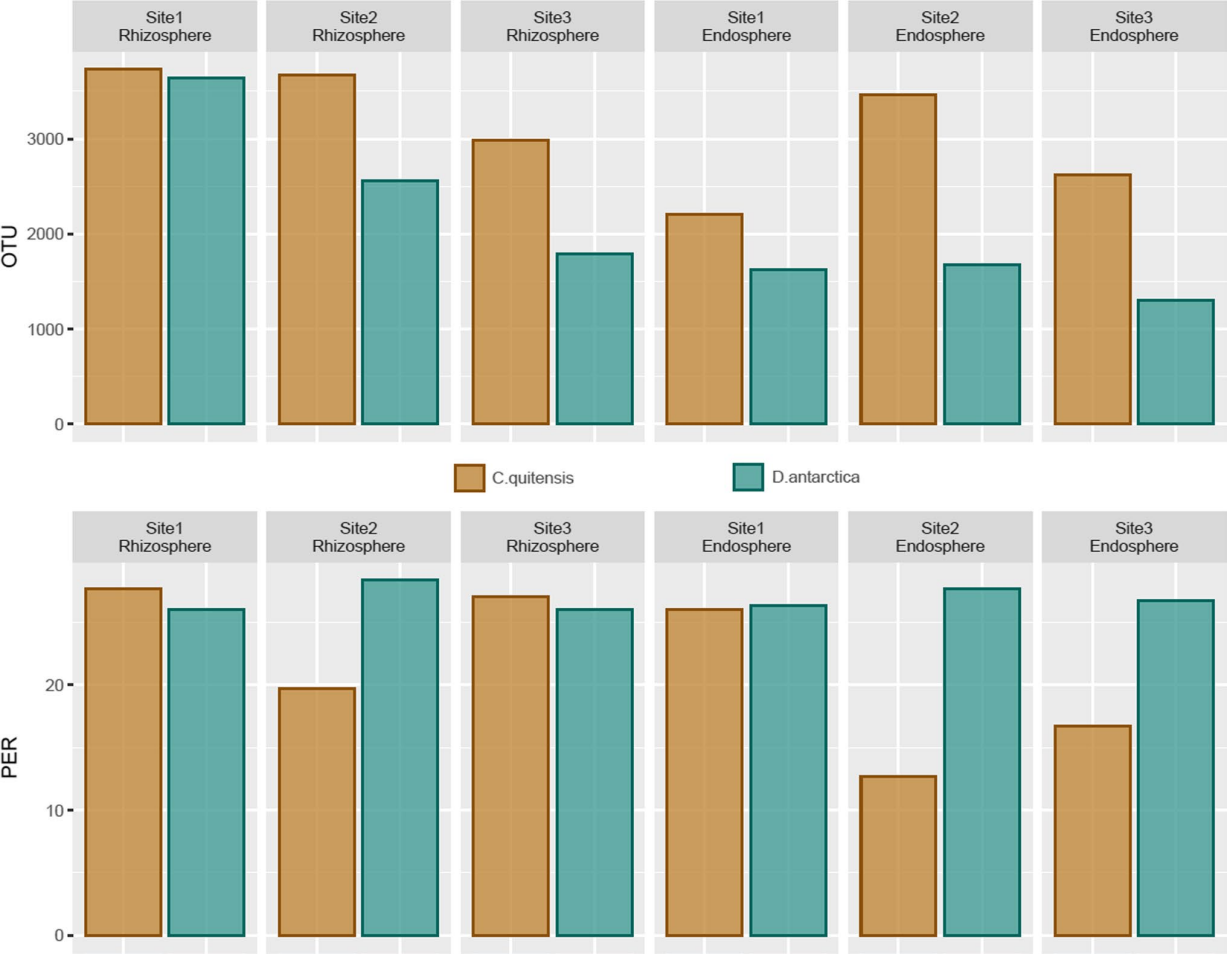
Operational taxonomic unit (OTUs) (upper row) and positive EcoPlate response (PER) numbers (lower row) for the bacterial communities associated with the rhizosphere and root endosphere of *Deschampsia antarctica* and *Colobanthus quitensis*

Bacterial operational taxonomic unit (OTU) numbers were always higher in the rhizosphere (av. 3064, sd. 778) compared to the root tissue (av. 2151.3, sd. 797.5). *C*. *quitensis*–associated communities had on average higher OTU numbers than *D*. *antarctica*–associated communities (soil, 3464.3 vs 2663.7; root, 2766.7 vs 1536). Rhizospheric communities from site 1 displayed the highest phylogenetic diversity for *D*. *antarctica* and *C*. *quitensis* alike (3637 and 3736 OTUs respectively), while the lowest values were noted for site 3 samples (*D*. *antarctica*, 1790; *C*. *quitensis*, 2987). *C*. *quitensis* endosphere community was least diverse in samples from site 1 (2210 OTUs) whereas for *D. antarctica* in samples from site 3 (1300 OTUs). The most diverse endospheric community was noted in site 2 samples for both plant species (*D. antarctica*, 1681 OTUs; *C*. *quitensis*, 3468 OTUs). Community response numbers on Biolog EcoPlates were on average lower in the endosphere compared to the rhizosphere for *C. quitensis* samples (av. 18.4, sd. 6.8 and av. 24.8, sd. 4.4, respectively), while for *D. antarctica*, those samples were comparable (rhizosphere, av. 26.78, sd. 1.3; endosphere, av. 26.89, sd. 0.7) (Fig. 2).

**Fig. 3:**
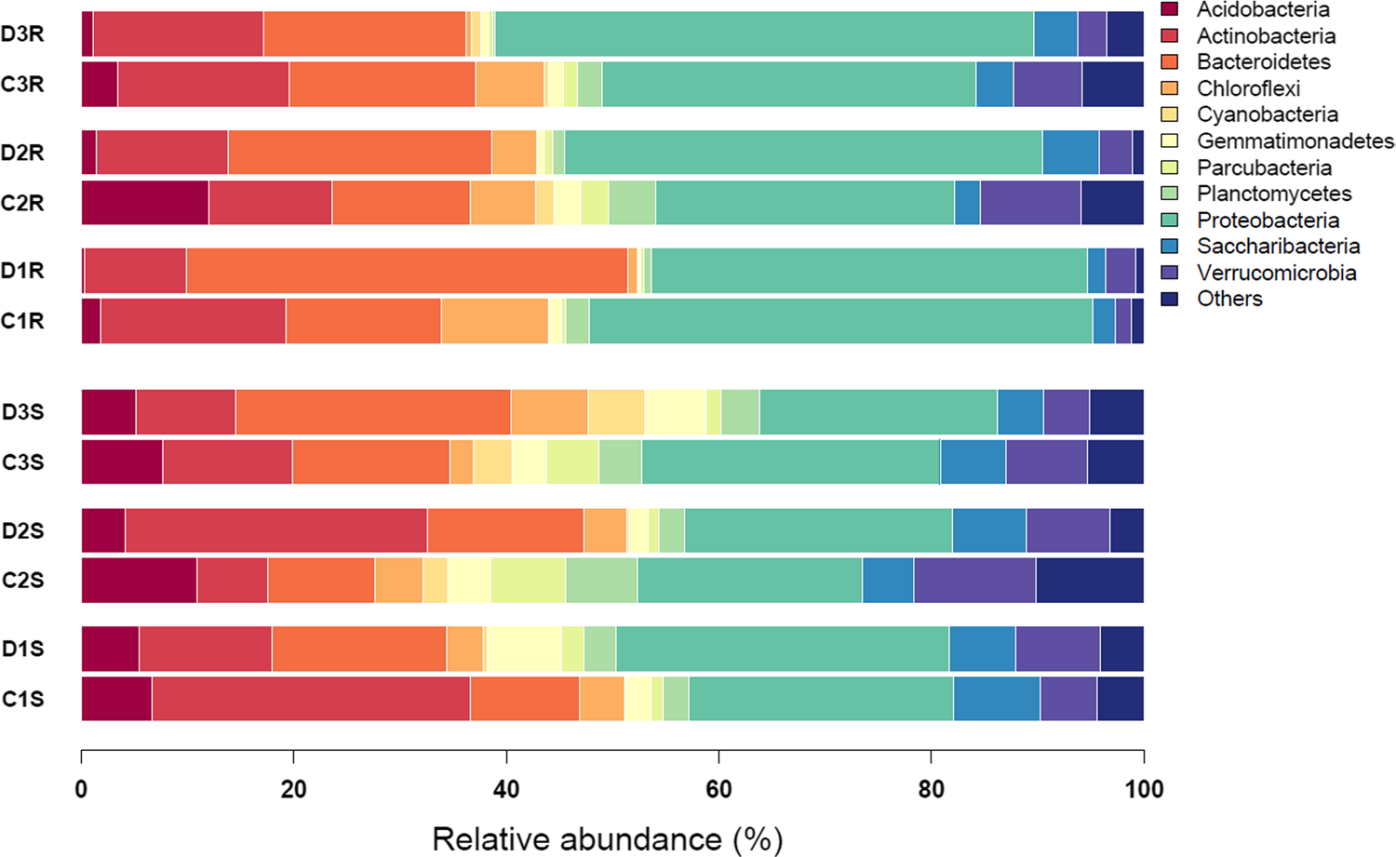
Relative abundance by percentile contribution of sequences identified on a phylum-rank taxonomic level. S, rhizospheric soil samples; R, root samples; D, *Deschampsia antarctica*; C, *Colobanthus quitensis*

The rhizosphere community of *C*. *quitensis* and *D*. *antarctica* was most abundant in the following bacterial phyla: *Proteobacteria* (av. 24.7%, sd. 3.4 and av. 26.3%, sd. 4.6, respectively), *Actinobacteria* (av. 16.2%, sd. 12.2 and av. 16.8%, sd. 10.2), *Bacteroidetes* (av. 11.7%, sd. 2.6 and av. 19.0%, sd. 6.0), *Saccharibacteria* (av. 6.4%, sd. 1.6 and av. 5.9%, sd. 1.4), *Verrucomicrobia* (av. 8.1%, sd. 3.1 and av. 6.7%, sd. 2.0), *Acidobacteria* (av. 8.4%, sd. 2.2 and av. 4.9%, sd. 0.7), and *Parcubacteria* (av. 4.4%, sd. 3.0 and av. 1.5%, sd. 0.6). The root endosphere of both plants was occupied largely by bacteria of the phylum *Proteobacteria* (*C*. *quitensis*, av. 36.9%, sd. 9.8; *D*. *antarctica*, av. 45.6%, sd. 4.9) but also by *Bacteroidetes* (*C*. *quitensis*, av. 15.1%, sd. 2.3; *D*. *antarctica*, av. 28.5%, sd. 11.7) and *Actinobacteria* (*C*. *quitensis*, av. 15.1%, sd. 3.1; *D*. *antarctica*, av. 12.7%, sd. 3.2). Considerable differences between plant species were noticeable for *Acidobacteria* (*C*. *quitensis*, av. 5.8%, sd. 5.5; *D*. *antarctica*, av. 0.9%, sd. 0.6) and *Planctomycetes* (*C*. *quitensis*, av. 3.0%, sd. 1.3; *D*. *antarctica*, av. 0.7%, sd. 0.4). The only significant (at *p* < 0.03) difference in relative abundance was noted for the *Chloroflexi* bacteria (*C*. *quitensis*, av. 7.5%, sd. 2.2; *D*. *antarctica*, av. 1.9%, sd. 2.0) (Fig. 3).

**Fig. 4:**
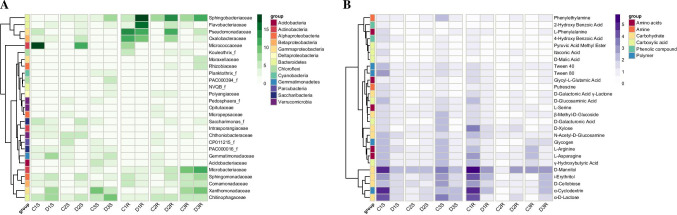
Heatmaps. **A** Sequence contribution identified on a family-rank taxonomic level (value according to sequence contribution percentage); **B** community responses on Biolog EcoPlates (mean *A*_590_ values from three replicates) S, rhizospheric soil samples; R, root samples; 1–3, sampling site numbers

Several bacterial families were present in considerable amounts in the rhizosphere, although the occurrence of some of them was highly site specific (Fig. 4A). Relative abundance of the family *Micrococcaceae* showed severe differences for *C*. *quitensis* (av. 6.3%, sd. 10) as well as for *D*. *antarctica* (4.6%, sd. 6.4), displaying highest values in site 1 for *C*. *quitensis* (17.9%) and in site 2 for *D*. *antarctica* (12.0%). High average abundance with concomitant high variations between samples was also observed for the family *Xanthomonadaceae* (*C*. *quitensis*, av. 4.7%, sd. 4.9; *D*. *antarctica*, av. 5.0, sd. 3.0). Relative abundance for this family peaked in site 3 for *C*. *quitensis* (10.4%) and in site 1 for *D*. *antarctica* (7.3%). Most stable levels of relative abundance were noted for the family *Chitinophagaceae* (*C*. *quitensis*, av. 4.5%, sd. 1.1; *D*. *antarctica*, av. 7.8%, sd. 1.6) and the candidate family (PAC000016_f) of the phylum *Saccharibacteria* (*C*. *quitensis*, av. 3.6%, sd. 0.1; *D*. *antarctica*, av. 2.7%, sd. 1.1).

The endosphere of both plant species was dominated by the family *Sphingobacteriaceae* (*C*. *quitensis*, av. 6.6%, sd. 0.7; *D*. *antarctica*, av. 14.9%, sd. 3.7). *D*. *antarctica* root endosphere also exhibited a high relative abundance of the family *Pseudomonadaceae* (av. 9.6%, sd. 3.3), while in *C*. *quitensis*, this family for noticeably represented only in site 1 (14.5%), accompanied by the family *Oxalobacteraceae* (12.7%). Similarly, the family *Flavobacteriaceae* occurred in considerable abundance in *D*. *antarctica* root endosphere only in site 1 (15.5%). Relative abundances of the family *Microbacteriaceae* was high in site 3 for both *C*. *quitensis* (8.6%) and *D*. *antarctica* (10.8%). A similar situation was observed for the family *Xanthomonadaceae* (*C*. *quitensis*, 7.7%; *D*. *antarctica*, 9.9%). *Chitinophagaceae* were present in all root samples at comparable levels (*C*. *quitensis*, av. 4.6%, sd. 1.1; *D*. *antarctica*, av. 4.2%, sd. 0.4). Noteworthy here are the relative abundances of alphaproteobacterial families. The *Sphingomonadaceae* were present in the roots of *C*. *quitensis* (av. 3.8%, sd. 2.3) and *D*. *antarctica* (av. 5.4%, sd. 1.6), while the *Rhizobiaceae* occupied only *D*. *antarctica* roots (av. 3.2%; *C*. *quitensis*, 0.4%).

Metabolic features of the rhizospheric community revolved mainly around carbohydrate catabolism (Fig. 4B). Highest absorbance value at 590 nm (*A*_590_) obtained for the rhizosphere was *A*_590_ = 4.93. The most actively catabolized compound was D-mannitol, both in *C*. *quitensis* (av. *A*_590_ = 3.6, sd. 1.4) and *D*. *antarctica* rhizosphere (av. *A*_590_ = 2.5, sd. 0.1). The glucose-containing polymer α-cyclodextrin was also readily metabolized (*C*. *quitensis*, av. *A*_590_ = 2.5, sd. 2.0; *D*. *antarctica*, av. *A*_590_ = 1.8, sd. 0.3), albeit for *C*. *quitensis*, its catabolism was most pronounced in site 1 (*A*_590_ = 4.7), similarly for α-D-lactose (*C*. *quitensis*, av. *A*_590_ = 3.0, sd. 1.8, site 1 *A*_590_ = 4.3; *D*. *antarctica*, av. *A*_590_ = 1.6, sd. 0.3). Other actively catabolized in the rhizosphere compounds included D-cellobiose (*C*. *quitensis*, av. *A*_590_ = 2.2, sd. 0.7; *D*. *antarctica*, av. *A*_590_ = 1.4, sd. 0.1), i-erythritol(*C*. *quitensis*, av. *A*_590_ = 2.2, sd. 0.9; *D*. *antarctica*, av. *A*_590_ = 1.7, sd. 0.3), L-asparagine (*C*. *quitensis*, av. *A*_590_ = 1.9, sd. 0.7; *D*. *antarctica*, av. *A*_590_ = 1.2, sd. 0.1), L-arginine (*C*. *quitensis*, av. *A*_590_ = 1.9, sd. 0.9; *D*. *antarctica*, av. *A*_590_ = 1.3, sd. 0.2). The endospheric community displayed similar features as the rhizospheric community. The highest absorbance value at 590 nm (*A*_590_) obtained for the endosphere was *A*_590_ = 5.86. The main difference between plant species was the more uniform catabolism intensity of carbon sources for *D*. *antarctica*, most notably for D-mannitol (*C*. *quitensis*, av. *A*_590_ = 3.4, sd. 2.4; *D*. *antarctica*, av. *A*_590_ = 2.8, sd. 0.04). Most high-absorbance values for *C*. *quitensis* were obtained for the samples from site 1: α-D-lactose (*A*_590_ = 4.53), α-cyclodextrin (*A*_590_ = 4.59), i-erythritol (*A*_590_ = 4.35), D-xylose (*A*_590_ = 3.54) and D-cellobiose (*A*_590_ = 3.17). The catabolism intensity of those carbohydrates was more uniform across the endospheric samples of *D*. *antarctica*: α-D-lactose (av. *A*_590_ = 1.6, sd. 0.4), α-cyclodextrin (av. *A*_590_ = 1.8, sd. 0.5), i-erythritol (av. *A*_590_ = 2.0, sd. 0.9), D-xylose (av. *A*_590_ = 1.4, sd. 0.1), and D-cellobiose (av. *A*_590_ = 1.6, sd. 0.4).

**Fig. 5:**
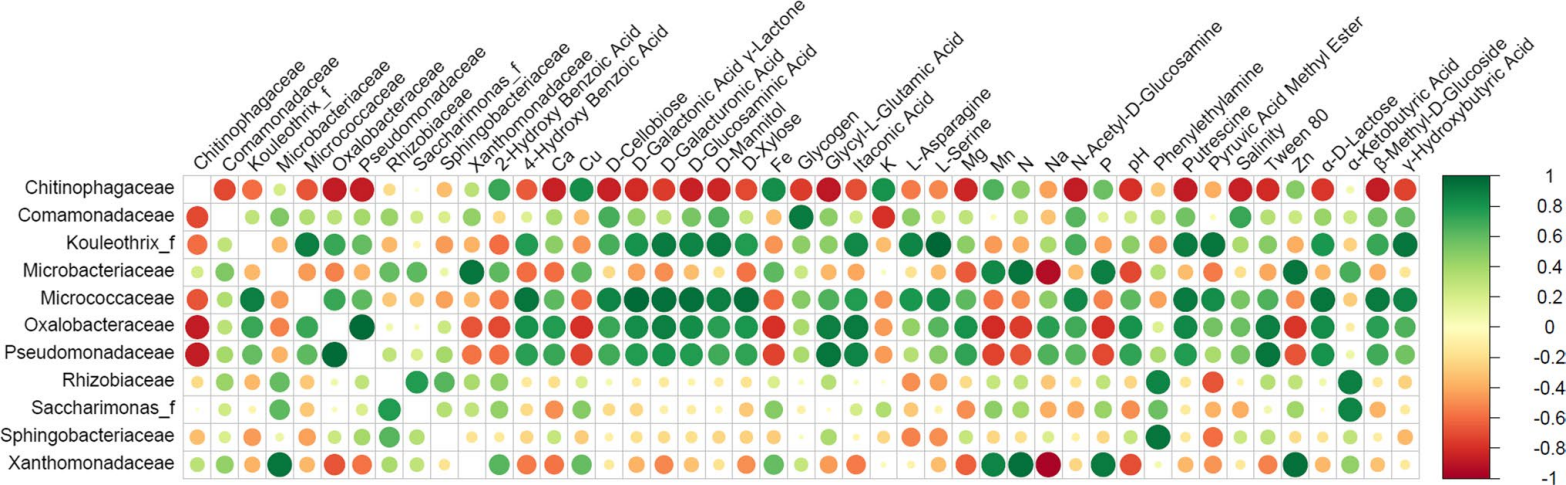
Correlogram of root endosphere family-rank sequence abundance, soil chemistry, and Biolog EcoPlate response data. Only significant (*p* < 0.05) correlations are shown

Several correlations in the root endosphere of both plants were apparent between biological and geochemical components (Fig. 5). The relative abundance of the family *Micrococcaceae* displayed significant correlations with the catabolism of several compounds, most notably D-xylose (*p* = 0.003) and α-D-lactose (*p* = 0.008). The *Pseudomonadaceae* and *Oxalobacteraceae* displayed significant correlations with the same compounds. Phenylethylamine catabolism was positively correlated with the occurrence of *Sphingobacteriaceae* (*p* = 0.008) and the *Rhizobiaceae* (*p* = 0.02). Negative correlations revolved mainly around the relative abundance of the family *Chitinophagaceae*. It displayed negative correlations with the catabolism intensity of several compounds, including D-cellobiose (*p* = 0.03) and D-mannitol (*p* = 0.04) but also with the geochemical parameters like salinity (*p* = 0.03) and calcium content (*p* = 0.03) and the relative abundance of other families like *Pseudomonadaceae* (*p* = 0.02) and *Oxalobacteraceae* (*p* = 0.03). It showed however positive correlations with heavy metal concentrations (Cu/Fe *p* = 0.04). *Microbacteriaceae* and *Xanthomonadaceae* displayed negative correlations with sodium contents (*p* = 0.007 and *p* = 0.002, respectively), while the *Oxalobacteraceae* showed negative relations with manganese concentrations (*p* = 0.047).

**Fig. 6:**
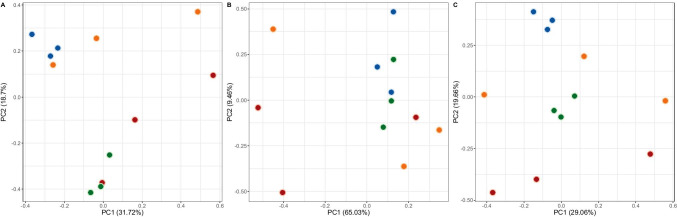
Principal component analysis (PCA) of biological data. **A** PCA based on percentage contribution of bacterial sequences identified on a family-rank level. **B** PCA based on responses obtained for bacterial communities by the Biolog EcoPlate method. **C** PCA based on a combination of family-rank bacterial sequence percentile contribution and normalized community responses on Biolog EcoPlates. Green dots, *Deschampsia antarctica* rhizosphere data; blue dots, *Deschampsia antarctica* endosphere data; red dots, *Colobanthus quitensis* rhizosphere data; orange dots, *Colobanthus quitensis* endosphere data

PCA showcased several phenomena within both plants’ root-associated microbial communities (Fig. 6). The PCA clustering based on the relative abundance of family-rank groups indicated that the rhizosphere community differs in structure from the endosphere community, both for *C*. *quitensis* and *D*. *antarctica*. The bacterial communities of *C*. *quitensis* showed great differences between sampling sites, while those of *D. antarctica* clustered according to the rhizocompartment of origin. The situation was similar for the EcoPlate-based clustering, mainly for *C*. *quitensis*, where no apparent clustering was observed. Rhizospheric and endospheric *D*. *antarctica* communities formed a loose cluster in this analysis. PCA based on a combination of phylogenetic and physiological data revealed a clear distinction between *C*. *quitensis* and *D*. *antarctica* root-associated communities. Two tight clusters emerged, separately harboring the rhizospheric community and the endospheric community of *D*. *antarctica*, while for *C*. *quitensis*, there were no apparent similarities between the samples.

**Fig. 7:**
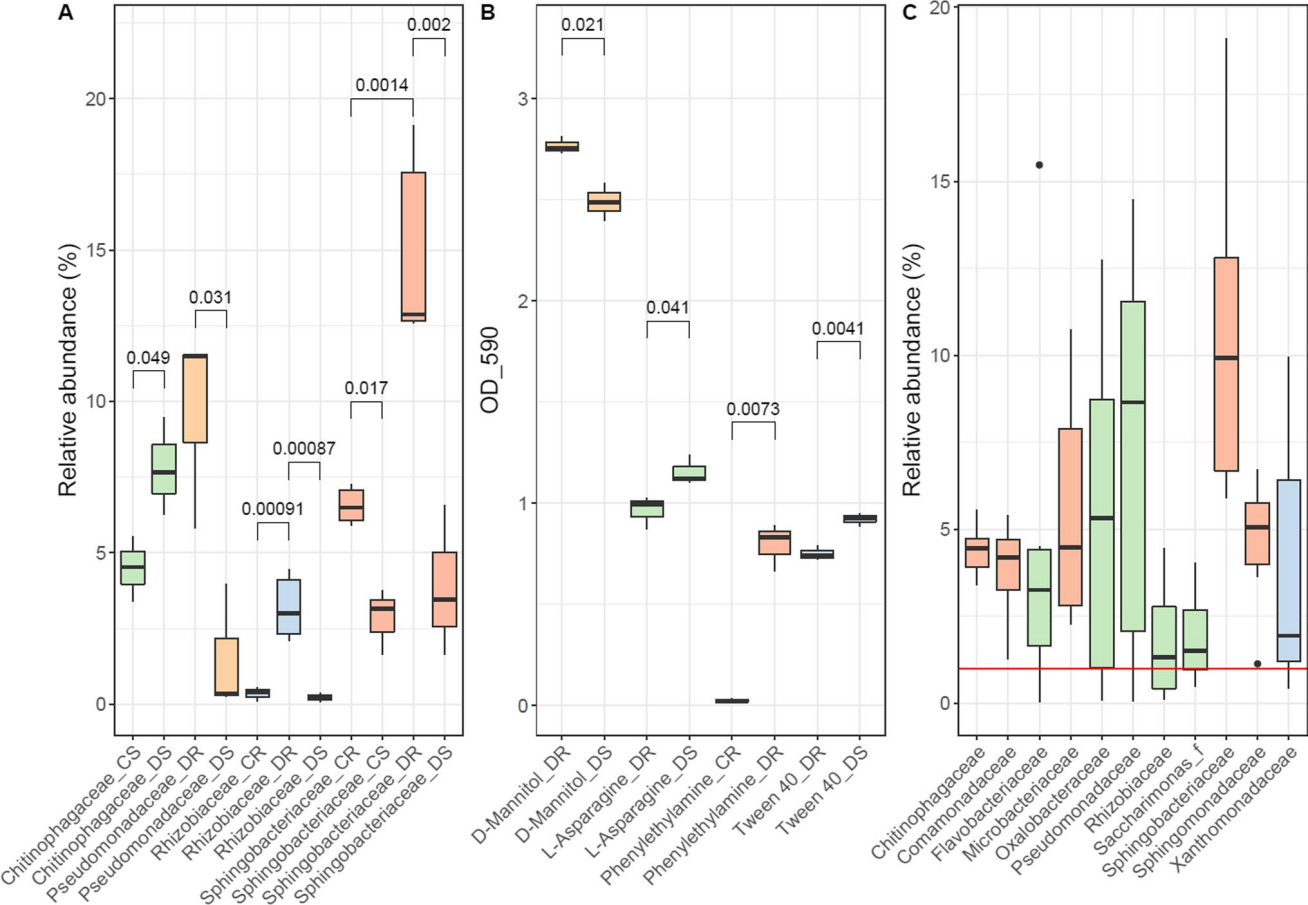
**A** Statistically significant differences (*p* < 0.05) within *Deschampsia antarctica* and *Colobanthus quitensis* rhizosphere/endosphere communities based on sequence contribution identified on a phylum taxonomic level; **B** statistically significant differences (*p* < 0.05) within *Deschampsia antarctica* and *Colobanthus quitensis* rhizosphere/endosphere communities based on community responses on Biolog EcoPlates; **C** core microbiome of *Deschampsia antarctica* and *Colobanthus quitensis* endosphere communities based on sequence contribution (> 1%, red line) identified on a family-rank taxonomic level. Red boxplots, bacterial families present in the roots of both plant species at > 1%; green boxplots, bacterial families present only in the roots of *Deschampsia antarctica* at > 1%; blue boxplot, bacterial family present only in the roots of *Colobanthus quitensis* at > 1%

Significant differences in phylogenetic and physiological levels emerged between microbial communities of *C*. *quitensis* and *D*. *antarctica* (Fig. 7 A and B). In the rhizosphere of *D*. *antarctica*, relative abundance of the family *Chitinophagaceae* was significantly higher (*p* = 0.049) than in the *C. quitensis* rhizosphere. The endosphere communities of *D. antarctica* were significantly richer in sequences of the family *Rhizobiaceae* (*p* = 0.00091) and *Sphingobacteriaceae* (*p* = 0.0014). Phenylethylamine catabolism was significantly more pronounced in the *D*. *antarctica* root endosphere (*p* = 0.0073).

The endospheric core community was based on the relative abundances of family-rank groups (Fig. 7C). Those that displayed an abundance of 1% or higher were scored as core community members. Only five families were at sufficient abundance in the endosphere of both examined plant species: *Sphingobacteriaceae* (5.9–19.1%), *Sphingomonadaceae* (1.1–6.7%), *Microbacteriaceae* (2.3–10.8%), *Chitinophagaceae* (3.4–5.6%), and *Comamonadaceae* (1.3–5.4%). Five families were above the 1% threshold only in *D*. *antarctica* endosphere samples: *Pseudomonadaceae* (*C*. *quitensis*, 0.1–14.5%; *D*. *antarctica*, 5.8–11.6%), *Oxalobacteraceae* (*C*. *quitensis*, 0.1–12.7%; *D*. *antarctica*, 2.2–8.9%), *Flavobacteriaceae* (*C*. *quitensis*, 0.03–4.1%; *D*. *antarctica*, 1.4–15.5%), *Sacharimonas* family (*C*. *quitensis*, 0.5–1.8%; *D*. *antarctica*, 1.2–4.0%), and *Rhizobiaceae* (*C*. *quitensis*, 0.1–0.6%; *D*. *antarctica*, 2.1–4.5%). Only one family, the *Xanthomonadaceae*, made the cut for *C*. *quitensis* (*C*. *quitensis*, 1.2–7.7%; *D*. *antarctica*, 0.4–10.0%).

## Discussion

A large body of literature dedicated to root-associated microbiomes indicates that bacterial and fungal communities dwelling in the plant rhizosphere and endosphere are host-specific species [39–41]. Nonetheless, the physiological status of this host plant influences the phylogenetic structure and metabolic capabilities of the associated microbiome [42]. This physiological status however is dependent on the edaphic and climatic conditions experienced by the plant [43].

Our results show that Antarctic flowering plants shape their root-associated microbiome differently, resulting in divergent microbial communities. The microbiome of *C*. *quitensis* root system bears different characteristics in each of the examined locations. In site 1 characterized by low essential nutrient concentrations (N-P-K) and high salinity and pH, the rhizosphere contained a highly diverse bacterial community, both phylogenetically and metabolically. The family *Micrococcaceae* (*Actinobacteria*) seemed to be a vital component of the rhizosphere in such conditions [44]. Members of this family along with other *Actinobacteria* have been observed in large quantities in salt marsh plants’ rhizosphere, indicating their stress alleviating effect in low water activity substratum [45, 46]. The corresponding *C*. *quitensis* endosphere was occupied by a fraction of metabolically active opportunitroph bacteria as indicated by the relatively low OTU numbers accompanied by high numbers of positive EcoPlate responses. Those were mainly the *Pseudomonadaceae* and *Oxalobacteraceae* family members. Inoculation with different *Pseudomonadaceae* strains has improved the salt-tolerance of *Zea mays*, which was connected to the water-binding exopolysaccharides produced by those bacteria [47], whereas the *Oxalobacteraceae* were enriched by the same plant species in nitrogen-poor soils, stimulating lateral root growth, consequently increasing nitrogen compound and other resources acquisition [48]. Furthermore, as these two families harbor mostly copiotrophic bacteria that display a multitude of metabolic features [49], their relative abundances in the endosphere were significantly correlated with the catabolism intensity of some of the carbon sources, most notably plant cell wall components: D-cellobiose and D-xylose. Cellulases and xylanases are essential in allowing bacterial entry into plant roots [50, 51]. At site 2, the structure of the root-associated communities diverges considerably from those at site 1. High phylogenetic diversity was accompanied by low metabolic activity and numbers of utilized carbon sources. This indicates that the community consists of either low activity bacteria or that respiratory activity was restricted at this location in *C*. *quitensis* rhizosphere. In tundra soils such as this one, nitrate or mineral nitrogen compounds may be deficient as most are bound in organic matter, which decompose extremely slowly under polar conditions [52, 53]. Some hypothesize that in this scenario, plants might exhibit microbivory by releasing proteases into the rhizosphere to liberate the microbe-bound nitrogen but also by destroying the cells of the endosphere microbes through oxidizing agent production on root cell plasma membranes [54]. Furthermore, the examined endosphere contained bacteria that are not usually found in this rhizocompartment, namely those belonging to the phylum *Acidobacteria* and *Gemmatimonadetes* [20]. In this regard, active endocytosis has been detected in *Arabidopsis thaliana*, which internalized and digested non-endophyte microbes [55]. At site 3, the root-associated microbiome of *C*. *quitensis* displays yet a different structure. This site is particularly rich in nitrogen, not only in the examined nitrates, but also in ammonia and organic forms [28]. Here the phylogenetic diversity in the rhizosphere community is relatively moderate, but the metabolic diversity is high, while the endosphere community displays moderate phylogenetic diversity and very low microbial activity. The predominant bacterial family in the rhizosphere were the *Xanthomonadaceae*, which displayed a positive correlation with soil nitrate contents. On several occasions, this bacterial group was observed to increase in numbers when organic and mineral nitrogen fertilization was applied [56, 57]. Together with the *Microbacteriaceae* and some other low abundance families, they constituted the bulk of the *C*. *quitensis* endosphere. The mentioned low activity of these bacteria might be due to host-defense mechanisms, enhanced by the heightened nitrate levels, as they were proved to promote defense signal molecules production like spermine and spermidine [58].

Based on the results of the PCA clustering, the microbial communities of *D*. *antarctica* displayed a high degree of similarity between samples within a particular rhizocompartment as compared to the vastly divergent *C*. *quitensis* communities. However, the microbiome of *D*. *antarctica* was also prone to restructuring enforced by the prevailing abiotic conditions. *D*. *antarctica* in the rhizosphere and the endosphere harbored in the majority of cases a phylogenetically low diversity community but highly active in terms of variety of catabolized carbon compounds. An exception was the salt-stressed site 1 community, where no specific bacterial group was enriched in the rhizosphere. However, in the endosphere of this site, *Bacteroidetes* families were strongly featured: *Flavobacteriaceae* and *Sphingobacteriaceae*. The latter was a consistent inhabitant of the *D*. *antarctica* endosphere, and their mean relative abundance was significantly higher compared to *C*. *quitensis* endosphere. Members of this family were noted to proliferate in salt-stressed plants’ rhizosphere and root tissues [59, 60] and were proven to confer tolerance to osmotically challenging conditions [61]. Those families were also observed in the invasive grass *Poa annua* L. communities presumably aiding its establishment in Antarctica, especially the *Flavobacteriaceae* [62]. An interesting case is the relative abundance of the family *Rhizobiaceae*, known to hold key species of plant beneficial rhizobacteria [63]. The occurrence of *Rhizobiaceae* and *Sphingobacteriaceae* was positively correlated with phenylethylamine catabolism intensity. For the *Rhizobiaceae*, this connection was previously described by [64] and was thought to indicate the formation of nitrogen-fixing bacteroids within the plant host cells. Despite some site-specific anomalies, the root-associated communities of *D*. *antarctica* displayed far greater stability across the sampling locations than *C*. *quitensis* communities. This could indicate that at least part of the *D*. *antarctica* root-associated microbial community is transmitted vertically, either by seeds or vegetatively due to the scattering of turf pieces. Monocotyledons of the *Poaceae* family were proven to be superior in carrying a beneficial bacteria load with their seeds as compared to other plant species [65]. This load of selected plant beneficial bacteria can be responsible for the ecological success of *D*. *antarctica* in the Antarctic region and the wider ecological niche than *C*. *quitensis* [66, 67] but also for its relatively low genetic diversity [15]. The variability that is introduced by sexual reproduction might diminish the grass’ compatibility with its associated microbial community which could have evolved since the Pliocene colonization event. Such compatibility loss was frequently observed in cultivars of genetically altered agricultural crops [68, 69]. While the majority of the *D*. *antarctica* root microbiome might contain facultative endophytes, dispersing through the soil and colonizing *C*. *quitensis* as indicated by the common core microbiome consisting of five bacterial families, this might not apply to the *Rhizobiaceae*. They seem to be exclusive *D*. *antarctica* obligatory root endophytes, as they were not observed in considerable abundance in the rhizospheric soils nor the endospheres of here examined *C*. *quitensis* specimens nor the invasive in Antarctica grass *P*. *annua* (yet still present in European *P*. *annua* samples) [62].

In conclusion, the Antarctic-native flowering plants display different strategies in assembling their root-associated microbiomes. *C*. *quitensis* seems to adjust its resident microbial community to the prevailing conditions even making use of microbivory, presumably due to the lack of associated efficient nitrogen fixers. *D*. *antarctica* on the other hand is inclined to rely on a fixed subset of bacteria that are presumably vertically passed to the daughter plant. This grass species holds to some obligatory nitrogen-fixing endophytes as well as other taxa that do not colonize *C*. *quitensis* roots, yet a shared core microbiome is likely to exist. Consequently, the “enigma” behind the presence of only two flowering plants in Antarctica might be strongly connected to their unique relationships with rhizospheric and root-dwelling bacteria.

## Data Availability

Illumina reads were deposited in the NCBI Sequence Read Archive (SRA) as BioProject PRJNA726953.
